# Familial adenomatous polyposis syndrome with colorectal cancer in two Nigerians: a report of two cases and review of literature

**DOI:** 10.11604/pamj.2018.30.6.14077

**Published:** 2018-05-04

**Authors:** Matthew Olumuyiwa Bojuwoye, Abdulfatai Bamidele Olokoba, Olumuyiwa Ayotunde Ogunlaja, Sulaiman Olayide Agodirin, Olatunde Kazeem Ibrahim, Kenechukwu Chukwuemeka Okonkwo, Aminu Mansa Aliyu

**Affiliations:** 1 Department of Medicine, University of Ilorin Teaching Hospital, PMB 1459, Ilorin, Kwara State, Nigeria; 2 Department of Obstetrics and Gynaecology, Bowen University Teaching Hospital, Ogbomoso, Oyo State, Nigeria; 3 Department of Surgery, University of Ilorin Teaching Hospital, PMB 1459, Ilorin, Kwara State, Nigeria; 4 Department of Pathology, University of Ilorin Teaching Hospital, PMB 1459, Ilorin, Kwara State, Nigeria

**Keywords:** Familial adenomatous polyposis syndrome, adenomatous polyps, colorectal cancer, gastrointestinal bleeding

## Abstract

Familial adenomatous polyposis syndrome is a rare condition characterized by the presence of numerous adenomatous polyps in the gastrointestinal tract and associated with risk for colorectal cancer. The disease is scarcely reported in Nigeria and this is the index report in Ilorin. Two cases were clinically diagnosed in our facility. They both presented with gastrointestinal bleeding and numerous rectal and colonic polyps were identified at colonoscopy. Histological examination of the polyps in both cases revealed features in keeping with adenomatous polyps. This report highlights the occurrence of this precancerous condition.

## Introduction

Familial adenomatous polyposis syndrome (FAPS) is a rare genetic disease that is characterized by the presence of hundreds to thousands of polyps in the colon and rectum. Untreated, it inevitably leads to cancer [1]. About 1% of all colorectal cancers (CRC) are caused by FAPS [2]. It is an autosomal dominant hereditary disorder and was first described by Virchow in 1863 [3]. The associated mutant gene was linked to chromosome 5q in 1986. The specific gene: Adenomatous polyposis coli (APC) gene which was discovered in the mid-1990s has 2,843 codons in 15 translated exons [3]. The APC gene codes for the APC protein that acts as a tumor suppressor, which means that it keeps cells from growing and dividing too fast or in an uncontrolled way [4]. Most FAPS patients have a family history of colorectal polyps and cancer but 25-30% of them are "de novo", without any clinical or genetic evidence of FAP in family members [3]. Most patients are asymptomatic for years until the adenomas are large and numerous, and cause rectal bleeding or anaemia, or cancer develops. Affected patients often develop CRC at a young age; usually in the fourth decade [3]. Attuenuated form of the syndrome arises from mutations of other germlines in the APC genes; such cases manifest fewer numbers of polyps ranging from 20-100. Although the attenuated form also supports natural progression to CRC, it occurs at later ages usually in the sixth decade. Gardner syndrome is a clinical variant of FAPS which manifests non-cancerous tumors of the skin, soft tissue, and bones in addition to colonic polyps. Worldwide, colorectal cancer (CRC) is the third leading cause of cancer death [5]. It is the third most common cancer in the United States and African-Americans have the highest incidence rate of CRC among the different races [5]. Previous reports suggest a low incidence of CRC in Africa but recent evidence from most sub-Saharan African countries has shown a sharp rise in the incidence of CRC [6]. Colorectal polyps, especially the adenomatous polyps, are generally accepted to be precursors to CRC. One major explanation proposed for the relatively low prevalence of CRC in Nigeria is the rarity of polyposis syndromes [7]. Reports of polyposis syndromes are extremely rare from sub-Saharan Africa. Two cases of FAPS with late presentation are reported here.

## Patient and observation

**Figure 1:**
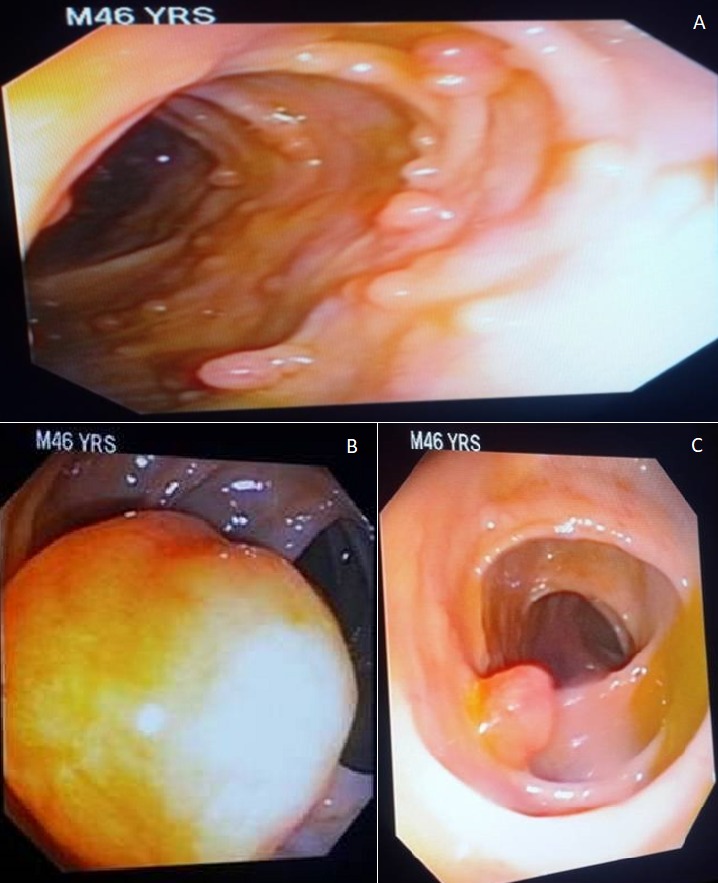
A) numerous polyps in the colon of case 1; B) a large polyp in the colon of case 1; C) the stump of a snared pedunculated polyp

**Case 1:** A 46- year old Nigerian male cleric presented to the Endoscopy suite of the University of Ilorin Teaching Hospital having been referred on account of an 8- month history of dyspepsia, progressive weight loss, diarrhea alternating with constipation, occasional haematochezia and painful peri-umbilical swellings. He had no fever, cough or drenching night sweats. No history of cigarette smoking or alcohol ingestion. He had a history of surgical removal of a mass lesion from his scalp 5 years earlier with a histological diagnosis of inclusion cyst. He had no known family history of either cancer or polyposis syndromes. He had a twin brother who died of an unknown cause in childhood. His other siblings were in good health. At presentation he was pale, had discrete non-tender right inguinal lymphadenopathy and Sister Mary Joseph (SMJ) nodules. Rectal examination revealed melaena. Gastroscopy revealed multiple sessile polyps in the antrum and a solitary polyp in the duodenal bulb. Histology of the gastric polyps revealed features of dysplasia. Colonoscopy performed using an Olympus CF-180 Evis Exera II forward-viewing colonoscope revealed hundreds of polyps of varying sizes extending from the rectum to the ascending colon. Two large polyps were seen at approximately 15cm and 90cm from the anal verge. Biopsies were taken from these, and one of the pedunculated polyps seen at 45 cm from the anal verge was snared (Figure 1 (A, B, C)). The histology of the large polyps was tubulovillous adenoma with low grade dysplasia whereas that of the snared polyp was tubular adenoma with low grade dysplasia. The histological diagnosis of the biopsies of the SMJ nodules was metastatic adenocarcinoma. He was commenced on chemotherapy.

**Figure 2:**
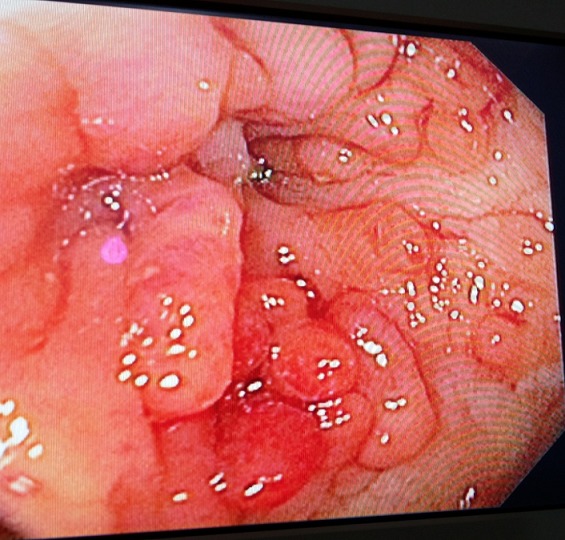
Numerous polyps in the colon of case 2

**Case 2:** A 49- year old native Nigerian male artisan (upholstery maker), was referred for colonoscopy on account of a 2-month history of colicky left lower abdominal pain, hematochezia and progressive generalized body weakness. He had no history of weight loss, passage of pellet like stool, spurious diarrhoea nor abdominal swelling. He had laparotomy done 24 years earlier on account of an acute abdominal condition. The details of the surgical findings were not known. He was neither hypertensive nor diabetic. He neither smoked cigarette nor drank alcohol. On examination he was pale and dehydrated. Pulse rate was 120 beats per minute and blood pressure was 120/70mmHg supine. No postural hypotension. Abdominal examination revealed hypertrophied right paramedian scar. His packed cell volume (PCV) was 20%, and he was transfused with two pints of fresh whole blood prior to colonoscopy with an Olympus CF-180 Evis Exera II forward-viewing colonoscope. Numerous sessile and pedunculated polyps of varying sizes were seen in the rectum and colon (Figure 2). Surgical intervention was delayed for about 3 months due to financial constraint and fitness concerns. The surgical findings were numerous polyps in the colon and rectum with a sigmoid mass lesion. Resection of the sigmoid mass along with some segments of the colon harbouring polyps was carried out. The histological diagnosis of the tumour was an adenocarcinoma. Post-operatively, he was commenced on chemotherapy.

## Discussion

The diagnosis of FAP is mainly by colonoscopy and the presence of 100 or more polyps that are later confirmed adenomas is clear indication of the diagnosis [8]. Double contrast barium enema is also very useful but lacks the opportunity to confirm that the polyps are adenomas. The two cases reported here are clinical variants of FAPS with late presentation. In the first case, the patient had more than 100 polyps of varying sizes in the entire colon with two large polyps believed to have undergone malignant transformation because of their endoscopic appearance, subsequent histological diagnosis of tubulovillous adenoma with dysplasia and the diagnosis of metastatic adenocarcinoma made from histology of biopsies obtained from the SMJ nodules. The presence of dysplastic gastroduodenal polyps further supports a clinical diagnosis of FAPS.

The second case was most likely a case of attenuated FAPS because the number of polyps viewed at colonoscopy was less than 100. The two patients presented with rectal bleeding which is the most common presentation in FAPS [9, 10]. First-degree relatives need rigorous screening and will need to have colonoscopy once every year. Screening should be carried out on children of affected parents, members of extended family who are at risk, and patients with suspected hereditary colon cancer. A family history of colonic polyposis could not be established in either of the two cases because we were unable to obtain voluntary consent from any of their relatives for screening colonoscopy. Noteworthy is the fact that in 20% of cases of FAPS, spontaneous mutations do occur without family history of polyposis syndrome or cancer [9]. Congenital hypertrophy of the retina pigment epithelium (CHRPE) was not present in both cases but this does not exclude the diagnosis of FAPS [9]. Neither molecular diagnosis nor genetic screening for the APC gene was carried out in these patients due to non-availability of the required screening tools in our facility.

## Conclusion

FAPS is rare in Africa where the incidence of colorectal cancer is reportedly low. These two case reports on two clinical variants of FAPS highlight the importance of this disease in our sub-region calling for a high index of suspicion to ensure early diagnosis and prevent the development of CRC.

## Competing interests

All authors declare that they have no competing interests related to this work.
